# Resistance is futile? Mucosal immune mechanisms in the context of microbial ecology and evolution

**DOI:** 10.1038/s41385-022-00574-z

**Published:** 2022-11-03

**Authors:** Emma Slack, Médéric Diard

**Affiliations:** 1 Laboratory for Mucosal Immunology, Institute for Food, Nutrition and Health, D-HEST, ETH Zürich, Zürich, Switzerland; 2 Botnar Research Institute for Child Health, Basel, Switzerland; 3 Biozentrum, University of Basel, Basel, Switzerland

## Abstract

In the beginning it was simple: we injected a protein antigen and studied the immune responses against the purified protein. This elegant toolbox uncovered thousands of mechanisms via which immune cells are activated. However, when we consider immune responses against real infectious threats, this elegant simplification misses half of the story: the infectious agents are typically evolving orders-of-magnitude faster than we are. Nowhere is this more pronounced than in the mammalian large intestine. A bacterium representing only 0.1% of the human gut microbiota will have a population size of 10^9^ clones, each actively replicating. Moreover, the evolutionary pressure from other microbes is at least as profound as direct effects of the immune system. Therefore, to really understand intestinal immune mechanisms, we need to understand both the host response *and* how rapid microbial evolution alters the apparent outcome of the response. In this review we use the examples of intestinal inflammation and secretory immunoglobulin A (SIgA) to highlight what is already known (Fig. 1). Further, we will explore how these interactions can inform immunotherapy and prophylaxis. This has major implications for how we design effective mucosal vaccines against increasingly drug-resistant bacterial pathogens

**Fig. 1:**
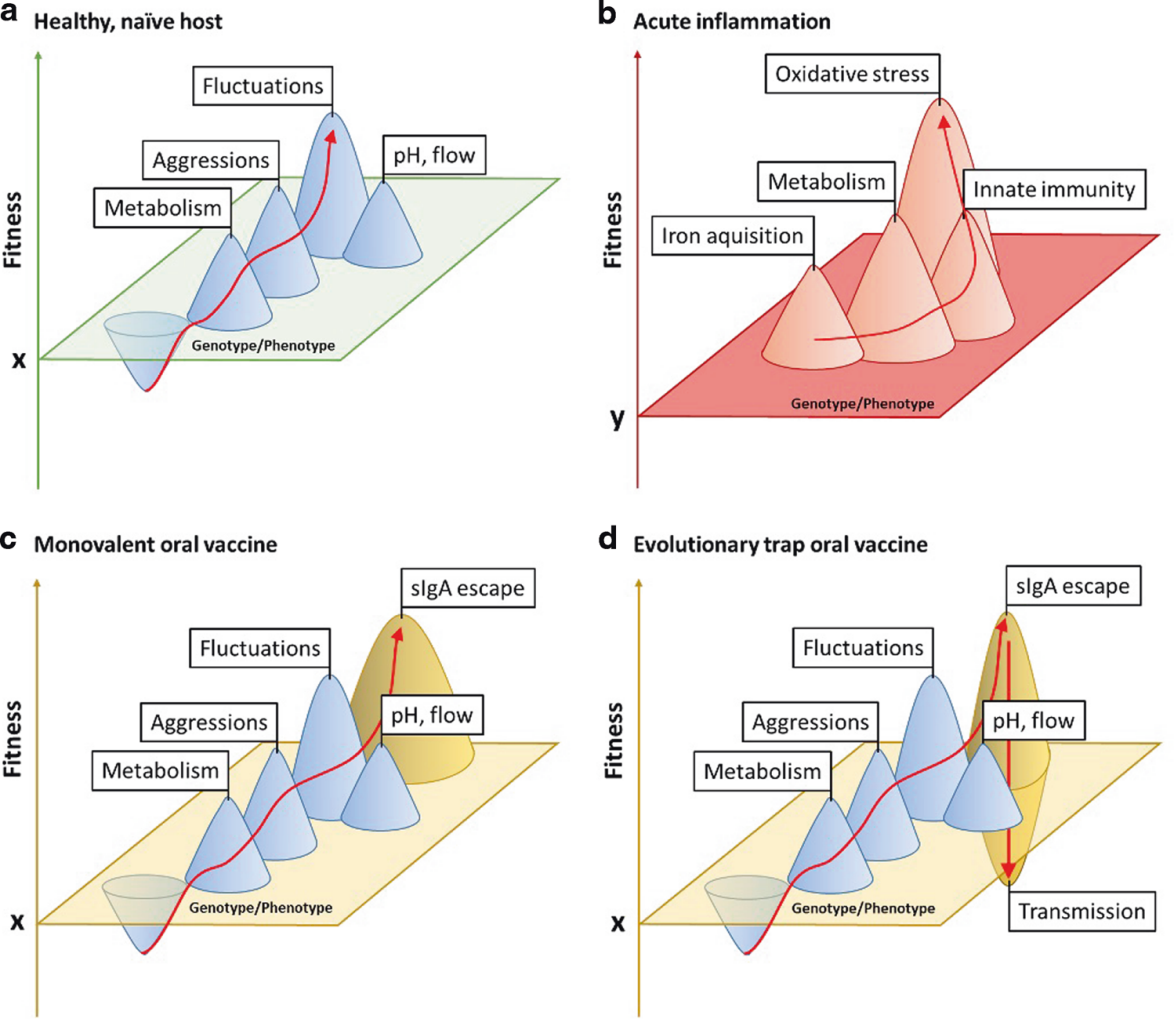
The immune response shapes the fitness landscape in the gastro-intestinal tract. The red arrows depict possible evolutionary paths of a novel colonizer along adaptive peaks in the intestinal fitness landscapes that change with the status of the host immune system. The flat surfaces represent the non-null fitness baselines (values x or y) at which a bacterium can establish at minimum carrying capacity. **a** In the healthy gut, metabolic competence, resistance to aggressions by competitors and predators, swift adaptation to rapid fluctuations as well as surviving acidic pH and the flow of the intestinal content, represent potent selective pressures and as many opportunities for bacteria to increase fitness by phenotypic or genetic variations. **b** When pathogens trigger acute inflammation, bacteria must adapt to iron starvation, killing by immune cells and antimicrobial peptides, and oxidative stress, while new metabolic opportunities emerge. **c** When high-affinity SIgA are produced against a bacterium, e.g., after oral vaccination, escape of SIgA by altering or losing surface epitopes becomes crucial for maximum fitness. However, escaping polyvalent SIgA responses after vaccination with “evolutionary trap” vaccines leads to evolutionary trade-offs: A fitness maximum is reached in the vaccinated host gut that represents a major disadvantage for transmission into naïve hosts (fitness diminished below x) (**d**).

The red arrows depict possible evolutionary paths of a novel colonizer along adaptive peaks in the intestinal fitness landscapes that change with the status of the host immune system. The flat surfaces represent the non-null fitness baselines (values x or y) at which a bacterium can establish at minimum carrying capacity. **a** In the healthy gut, metabolic competence, resistance to aggressions by competitors and predators, swift adaptation to rapid fluctuations as well as surviving acidic pH and the flow of the intestinal content, represent potent selective pressures and as many opportunities for bacteria to increase fitness by phenotypic or genetic variations. **b** When pathogens trigger acute inflammation, bacteria must adapt to iron starvation, killing by immune cells and antimicrobial peptides, and oxidative stress, while new metabolic opportunities emerge. **c** When high-affinity SIgA are produced against a bacterium, e.g., after oral vaccination, escape of SIgA by altering or losing surface epitopes becomes crucial for maximum fitness. However, escaping polyvalent SIgA responses after vaccination with “evolutionary trap” vaccines leads to evolutionary trade-offs: A fitness maximum is reached in the vaccinated host gut that represents a major disadvantage for transmission into naïve hosts (fitness diminished below x) (**d**).

## Bacterial fitness landscapes?

For bacteria growing in controlled environments, a useful (if imperfect) analogy to understand evolution is the “fitness landscape”. This reduces the multi-dimensional genotype-environment selective space to one or two dimensions, with fitness indicated on the vertical axis^1,2^. On this landscape, population evolution can be visualized as movement towards one or more fitness maxima: i.e., equilibria around which all further genetic changes are neutral or detrimental (Fig. 1). Using “tame” lab bacteria grown in flasks, the strongest influences on the fitness landscape are efficiency of nutrient uptake and usage, and the relative stresses exerted by expressing these uptake and metabolic systems. Nevertheless, very long-running experiments such as the Lenski “Long-term Evolution Experiment”, growing a clonal *Escherichia coli* (*E. coli*) population in a minimal media containing limiting amounts of glucose, reveal how diverse the selected outcomes can be even in apparently simple environments^3^.

It should then be noted that the gut lumen has only very limited similarity to a simple culture flask. The gastro-intestinal (GI) tract is an open system with regular, but temporally spaced, delivery of nutrients, distinct physiologies from mouth to anus, and non-uniform turbulent mixing/bulk flow of intestinal content. Gut motility, intestinal secretions, food intake and cecal pH show pronounced circadian rhythms^4,5^, which in turn have a major influence on the replication and metabolism of intestinal microbes^6–8^. The composition and density of the microbiota varies more or less continuously from mouth to anus, and also between mucus-layer associated bacteria and gut luminal populations^9,10^. The rate of flow in the intestine is also often underestimated by humans, as our sigmoid colon acts as a collecting vessel for continuously produced fecal material. In fact, the adult human intestine handles in the order of 10L of fluid per day, of which 8L are intestinal secretions that are re-absorbed along the GI tract^11^. As the internal diameter of most of the small intestine is less than 1 cm, this results in very fast flow rates. In contrast, in the large intestine the diameter swells to 5–10 cm and a large fraction of fluid has already been resorbed in the small intestine. Correspondingly, the flow rate in the large intestine is much lower. Nevertheless, calculations based on the water content of cecal and feces content in mice indicate that the upper large intestinal content turns over 2–3 times per day^12^. In the absence of very strong adhesion to epithelial surfaces, any bacterial species replicating more slowly than this in the large intestine will be diluted to extinction by flow alone^13,14^. Therefore, the gut lumen is a highly dynamic environment. Fitness maxima are expected to shift over the course of a day at any one point in the gut, as well as from the perspective of an individual bacterium making its journey along the GI tract^15^. Therefore, it is important to understand that our gut fitness landscape is rather elastic and is a 2D representation of a very high-dimensional space.

The achievable fitness of a bacterium depends not only on the fitness landscape, but also the speed with which a bacterium can adapt within that landscape relative to the stability of the fitness peaks. Evolution within fluctuating environments has been studied extensively in environmental ecology^16^ and in vitro systems^17^ as well as more recently in the mouse intestine^18,19^. The speed of adaption depends on (1) the mutation supply rate, defined as Ne*U, where Ne is the effective population size and U is the rate of accumulation of beneficial mutation per generation^20^, (2) the nature of the genetic changes required, and (3) the relative benefit of the acquired phenotype.

### Adaptation in a fluctuating fitness landscape

Mutations fuel adaptation. The mutation supply rate (Ne*U) varies with the effective bacterial population size, its rate of replication and its mutation rate per generation. Changes in the environment that either drastically reduce population size (Ne), or that alter replication rates, will therefore affect the evolvability of a bacterium, that is, the likelihood that maximum fitness will be reached within a given timeframe. To use the well-trodden metaphor: The more monkeys you have, and the faster they type, the higher the probability that one will produce the complete works of Shakespeare, before we decide we actually wanted to read Jane Austen.

The complexity of changes needed to reach a higher fitness equilibrium is also a major determinant of how likely the phenotype is to emerge. For example multiple mutations and/or epistasis may be involved, and “valleys” in the fitness landscape will select against some trajectories, However, horizontal gene transfer can allow bacteria to “jump” to new zones of the fitness landscape, for example opening new niches in the host^21^. A central tenant of the large intestinal microbiota is that it is highly abundant, actively growing and is in an ideal environment for horizontal gene transfer of plasmids and bacteriophages^22^. We can therefore assume that most microbes reach a fitness maximum within a relatively short window of intestinal colonization^23^. Indeed, adaption to the host environment can occur within days to weeks of intestinal colonization, especially when selective pressures are strong^23–29^. This is consistent with a high mutation supply rate, Ne*U, in the gut lumen.

It is then important to understand the (slightly counter-intuitive) conflict between mutation supply rate and fixation of mutants within a population. Once a beneficial mutation emerges in a population, it remains far from certain that it will reach fixation (100% in the population). The probability that such a mutant escapes stochastic loss depends on (1) the clearance rate and bottlenecks experienced by the population, (2) the relative benefit inferred by the mutation and (3) complex stochastic effects of clonal interference^19^. When the mutation supply rate is low, the chance to produce a mutant with increased fitness during a given timeframe is very low. When such a clone appears then it has a monopoly and there is a high probability that this mutation will become fixed: a process referred to as a hard selective sweep. However, when the mutation supply rate is high, as predicted in the gut, the chance to simultaneously generate several mutants with increased fitness is correspondingly higher. In this case, clonal interference, i.e., competition between these clones, decreases the likelihood that any one of these mutations completely takes over the population: a process referred to as a soft selective sweep. The likelihood of soft selective sweeps is further increased as adaptation is often constrained: The existence of valleys in the fitness landscape that select against some evolutionary trajectories can favor the generation of different competing mutants acquiring the same adaptive phenotype^23,25^. Thus, clonal interference and soft selective sweeps are expected to be the norm in the gut^19,30^.

Adaption is also influenced by the stability of the environment. A key feature of the GI tract is that gut environmental conditions fluctuate both over the circadian cycle, and over periods of days to weeks. These changes are driven by shifts in diet, antibiotic treatment, infection^18,19^ etc. Mutations that are beneficial at a given time-point can become detrimental when conditions change^24,26^. When fluctuations are fast and frequent, mutation accumulation and HGT may not be fast enough to avoid extinction. It is therefore both expected and observed that swift adaptability is highly beneficial in the gut^15^. Therefore both environment sensing, which fine-tunes gene expression, and stochastic switching are common features needed to reach a high fitness equilibrium in the gut^26,27,31–33^.

## The fitness landscape of the healthy intestine

Major determinants of the “fitness landscape” encountered by an individual bacterium invading into a gut microbiota community include:

- Availability of a metabolic niche, driven by diet as well as positive/negative metabolic interactions with other microbiota members^30,34–36^.
- Presence of a growth-permissive environment, e.g., pH and osmolarity, driven by flow rates, water handling, intestinal secretions and food components^14,13,37,38^.
- Aggression: Specific bacteriophages, type VI secretion systems, colicins etc: determined by composition of the rest of the microbiota^39–41^. Antimicrobial peptides, bile acids, digestive enzymes, immune effectors: determined by the host^42–44^.

These can be large effects—if a bacterium entering the GI tract has no access to carbon, or does not encounter a pH permissive for its growth, the presence/absence of an immune response will have little additive effect on its fitness. Alternatively, if a bacterium is close to its fitness optimum in the healthy intestine, then in order to see a major change in the fitness of the bacterium, any immune response or intervention needs to be sufficiently large to shift or remove this fitness maximum (Fig. 1).

Therefore, some major determinants of the healthy intestine fitness landscape for a particular microbe are controlled by host physiology and behavior i.e., are evolvable traits of the host. In order to increase host fitness, these traits should evolve under selective pressures to prevent colonization by strongly pathogenic species and conversely to promote colonization by benign/beneficial species capable of conferring metabolic benefits and outcompeting potential pathogens. In line with this, human milk oligosaccharides increase fitness of beneficial microbes in the neonatal microbiome^45,46^. In the adult gut during fasting, or when the diet lacks complex carbohydrates, it also appears that mucin-derived glycans provide a benefit to microbiota species that improved energy recovery from dietary fiber, whenever this is available^15,47–49^.

Nevertheless, there remains a large stochastic component to the fitness landscape based on the identity of the microbes^50^. Microbiome composition varies extensively from individual to individual and over time. Correspondingly, microbe-microbe and microbe-host interactions which affect the fitness landscape of an incoming bacterium vary between individuals and over time^51–53^. This coopting of the gut microbiota via host physiology is essential for the healthy gut to suppress pathogenic bacterial colonization – a phenomenon known as colonization resistance^54–56^. It is clear that germ-free mice and humans with microbiota disruption are very readily colonized by pathogens that can gain no foothold in “normal” individuals^54–56^. From this perspective, a simple definition of a “healthy” microbiota is one that minimizes the fitness of potential pathogens/pathobionts, in a way that is robust to typical daily perturbations. How this is achieved remains a very active area of research, that needs to move beyond simple metrics such as diversity to elucidate functional mechanisms^56–60^. In a good example of such progress Erble et al. demonstrated that a commensal *E.coli* can dramatically reduce the available niche for *S*. Typhimurium in the gnotobiotic mouse intestine by efficiently competing for galactitol as a carbon source^56^. Increasing evidence is also emerging on the role of microbe-microbe interactions in altering the immunogenicity/pro-inflammatory potential of other species^61^, demonstrating that these interactions do not only exclude or maintain particular species/niche, but can also modify the behavior of other species present.

## How the host immune system influences fitness landscapes and bacterial within-host evolution: (1) Inflammation

The potential impacts of inflammation on the gut environment are summarized in Table 1 below, but overall the message is clear: Acute inflammatory responses dramatically alter almost all aspects of the intestinal fitness landscape, ranging from nutrient availability through environmental conditions to inter-and intra-kingdom aggression. Unsurprisingly therefore, acute inflammation in the gut drives massive shifts in microbiota composition and abundance by remodeling the fitness landscape for every microorganism present.

**Table 1:** Shifts in the bacterial fitness landscape driven by acute inflammation.

Pathophysiology	Effect	References
Reactive oxygen and nitrogen species abundance	Selection of species capable of using nitrate/nitrite as electron acceptors. Selection for detoxification of reactive oxygen species. Selection for oxygen tolerance.	^68,131–134^
Antimicrobial peptides	Selection of species with intrinsic or acquired resistance to antimicrobial peptides. Differences in biogeography.	^69,135,136^
Simple metabolite availability	Benefit for fast-growing metabolic generalists	^67,137,138^
Neutrophils in the gut lumen	Pleotropic and redundant effects of enzymes, extracellular DNA, Hypochlorous acid. Strong bactericidal effects against almost all gut microbial species.	^66,139^
Altered luminal volume and flow rate	Wash-out of slow-growing, non-adhesive strains	^13^

While we like to think of our immune system as being genuinely useful, as in many situations in life, intestinal inflammation actually seems to be something of a trade-off. It is clearly necessary to control tissue loads of invasive bacteria, parasites and viruses—failure to do so is lethal^62–64^. But out in the gut lumen the benefits often appear to be more on the side of the pathogens and/or their mobile genetic elements. This has been well studied in the case of non-Typhoidal *Salmonella* infections and *Clostridioides difficile* infections^27,65–70^.

In models with major microbiota disruption, the main replicative site for non-Typhoidal *Salmonella* strains, as well as the source of bacteria transmitting to new hosts is the gut lumen^71^. Tissue invasion, driven by virulence factors encoded within the *Salmonella* Pathogenicity Island 1, actively induces inflammation in the large intestine which suppresses re-growth of the microbiota^27,72^. While in vitro systems have identified a range of possible immune mechanisms that could contribute to gut luminal clearance^73–76^, knock-out mice indicate high levels of redundancy in these systems. However, activated neutrophils in the gut lumen are potent contributors^66^. Situations in which neutrophils are abundantly recruited into the gut lumen drive 100–10,000-fold contractions of *Bacteroides thetaiotaomicron*^12^ and *S*. Typhimurium^66^ in the mouse gut lumen. Neutrophils release a broad range of antimicrobial compounds, as well as DNA in the form of neutrophil extracellular traps (NETs) and reactive oxygen and nitrogen compounds that can be used in metabolism by some bacterial species^67,68^. By disrupting established trophic chains in the gut, directly killing microbiota members, and selecting for bacteria resistant to high levels of reactive oxygen and nitrogen species, the landscape shifts in favor of facultative anaerobes able to use a broad range of simple carbon sources^67,68^.

This process has knock-on effects for the lysogenic bacteriophage carried in the genomes of most bacteria. In vitro, the most effective way to induce a genome-integrated lysogenic phage to enter the lytic cycle (i.e., to excise from the host genome, replicate, kill its host and release infectious phage particles) is to treat its host cells with DNA-damaging agents such as UV light or mitomycin C^77^. Much like rats leaving a sinking ship, many mobile genetic elements have evolved mechanisms to sense when their host may no longer be a safe-haven^78^. In the gut, bacterial SOS responses can be induced by the host inflammatory response – for example by damage to bacterial cells caused by reactive oxygen and nitrogen species^79^. Stress-induced phage lysis can *theoretically* be beneficial to the host if the stressed bacterium happens to be a pathogen, although this is rarely (if ever) directly measured. Additional selective pressure via the action of the released phage might also shift the fitness landscape for closely related bacteria in the gut due to direct infection^33^ or skewing towards an anti-viral host inflammatory response^80^. On the other hand, phage mobilization increases horizontal gene transfer in the gut, both of phage-borne accessory genes (morons) and via generalized transduction^22,79,81^. Phage morons can increase the intestinal fitness of pathogens facing innate and adaptive immune responses (e.g., Superoxide dismutase on phage Gifsy-2^82^, glucosyltransferases on phage remnants and P22 in *S*. Typhimurium^26,83^).

There are also knock-on effects of inflammation on plasmid-mediated horizontal gene transfer. In its simplest incarnation, the inflamed gut environment typically increases the niche, and therefore population density, for facultative anaerobes of the *Enterobacterales* family. The likelihood of cell-cell contact increases proportionally to the square of the cell density^84^, thus increasing plasmid exchange between these species^85^. This can accelerate the evolution of antibiotic resistance often encoded on plasmids in *Enterobacterales*.

In conclusion, the consequences of inflammation (including constriction of gut luminal volume, increase flow rates, production of reactive oxygen and nitrogen intermediates, increased bile and antimicrobial peptide concentration etc) on the gut bacterial fitness landscape are equivalent to a massive earthquake. In most cases, there are apparently detrimental effects on the fitness of beneficial microbiota members and increased horizontal gene transfer. Nevertheless, there is a lot of unexplored subtlety: not all gut inflammatory responses are equal either in the mechanisms induced, nor in their magnitude. How different grades and flavors of inflammation interact with diverse starting microbiota is potentially a major determinant of microbiota-associated diseases.

## How host immune system influences fitness landscapes and bacterial within-host evolution: (2) Secretory Immunoglobulin A

**Fig. 2:**
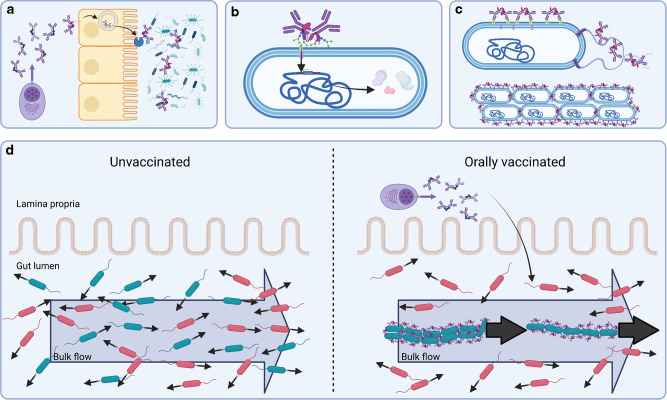
Mechanisms of SIgA-bacteria interactions in the GI tract. **a** IgA secretion across the intestinal epithelium by the Poly Ig Receptor. **b** Non-canonical interactions via O- and N-glycan binding and uptake, driving expression of polysaccharide utilization loci (PUL). **c** Canonical interactions inhibiting function or driving aggregation via enchained growth or classical agglutination. **d** Influence of aggregation on clearance due to flow of gut content. Figure generated with Biorender.

Secretory antibodies are specialized, typically multimeric, isotypes that are actively secreted across mucosal membranes. In mammals, birds and most reptiles, IgAs are the main secreted isotypes, with some species producing up to 15 different IgA heavy chains (e.g., rabbits) while humans produce IgA1 and IgA2, and mice produce only one (referred to just as IgA). The PolyIg Receptor (PIgR) binds and becomes covalently crosslinked to dimeric or multimeric IgA produced by plasma cells on the basal side of epithelial cells. This complex then transcytoses to the apical membrane where it is then cleaved, releasing a complex between multimeric IgA and the extracellular domain of PIgR, onto the mucosal surfaces^86–89^ (Fig. 2a). This complex is referred to as secretory IgA (SIgA).

Although monomeric IgA in the serum can activate innate immune mechanisms^90^, SIgA neither fixes Complement, nor interacts with any known activatory Fc receptors^91^. This has been a source of puzzlement: why do we produce such large quantities of an antibody isotype that does not appear to do anything? Based on the above discussion about the influence of intestinal inflammatory responses on bacterial selection in the gut, the uncoupling of intestinal antibodies and inflammation perhaps starts to make sense. In the context of complex bacterial consortia, acute inflammation disrupts the ecosystem—an activity that should be carried out only in cases of dire need. But if it is not killing bacteria, then what is SIgA actually doing?

Recent progress has started to shed light on SIgA mechanisms of action. These can be roughly divided into non-canonical interactions of bacteria with glycans/IgA-binding proteins, and canonical, specific interactions dependent on the antibody complementarity determining regions^91^. In comparison to the earthquake-like fitness landscape effects of inflammation, those of SIgA are more in the realm of controlled landscaping. This is not to say that the effects are weaker—we observe very strong IgA-mediated selective pressures^26^ but we would argue that they are more specifically manipulative.

### Non-canonical interactions

Interactions of gut bacteria with abundant O- and N-glycans decorating the secretory component, J-chain and antibody hinge regions has been linked to host-glycan foraging^92^. This was elegantly demonstrated by Nakajima et al, by adoptively transferring an ovalbumin-specific IgA to mice and tracking its effects on colonization of *Bacteroides thetaiotamicron*^92^. This upregulated the expression of several host-glycan-active “polysaccharide utilization loci” in the bacteria, with effects on overall community composition and stability. As SIgA is abundant in the gut lumen, SIgA glycan foraging can therefore generate positive selection for bacteria capable of metabolizing host O- and N-glycans (Fig. 2b).

### Canonical interactions

The fitness effect of SIgA canonical binding is expected to depend heavily on the affinity of antibody-bacteria interactions, as well as the specific target recognized. Very low-affinity interactions are not expected to bother the targeted bacteria^93^. In contrast high-affinity antibodies can profoundly alter fitness via both direct and indirect mechanisms. This can occur via several fundamentally different mechanisms:

### *Negative selection via neutralization*

Theoretically, SIgA could bind with sufficiently high affinity to neutralize functional surface molecules such as importers, adhesins or secretion systems of targeted pathogens. If the inhibited mechanism is non-redundantly required for bacterial growth in the gut, then this can directly impact gut colonization. Perhaps the best-documented example of this is neutralizing IgA to cholera toxin^94^, which is one component of the IgA response induced by the oral cholera vaccines. Cholera vaccination provides a sufficiently strong negative effect on fitness that *Vibrio cholera* fails to colonize to disease-causing levels in most vaccinated individuals—however this clearly requires more than just toxin neutralization^95,96^.

There are also reports of monoclonal SIgAs capable of inhibiting the function of individual bacterial proteins, i.e., “neutralizing” a specific function such as an outer-membrane porin or flagella, rather than a whole organism^97^. Effects of such antibodies can be seen in the bacterial transcriptome but may have negligible effects at the level of total population size.

### *Negative selection via enchained growth*

More frequently, protective SIgA responses, induced by oral vaccination or infection, are not neutralizing in any classical sense. Rather they target abundant bacterial surface glycans such as O-antigens, teichoic acids or capsular polysaccharides, or bind to outer-membrane proteins in a non-neutralizing manner^84,97–100^. Here, the protective mechanisms rather relate to cross-linking of bacteria and/or bacterial surface structures.

A very simple mechanism by which high-affinity surface-targeting SIgA can alter the fitness landscape in the gut is via bacterial aggregation^84^. This can happen via classical agglutination (collision, leading to SIgA cross-linking of identical bacteria^101^) or enchained growth (cross-linking of identical bacteria during cell division)^84^ (Fig. 2c). A handy analogy to understand how this aggregation alters fitness is to imagine yourself arriving on a train at a busy train station. There is typically bulk (but slightly turbulent) flow of people away from arriving train to the main station hall. Now imagine you are traveling with thirty, three-year-old children. If you simply released the children from the train onto the platform, you’d be rather surprised to find all of them back in the main station hall—at least a few of them will do random walks and will distribute across all available space. So instead, you ask the children to hold hands, two by two, in a “crocodile” which will move *en bloc*. SIgA-mediated aggregation achieves a similar feat for bacteria out in the gut lumen—aggregates forming far away from the intestinal epithelium are moved and lost en bloc in the fecal stream^84^. Simply put, clearance in flow becomes more efficient (Fig. 2d). This is a fitness disadvantage, even if growth and killing of the bacterium are unaffected. Correspondingly, SIgA-mediated aggregation selects *for* bacteria that compete for the same niche but escape SIgA cross-linking. One possible outcome is outgrowth of IgA escape-mutants of the targeted strain^26^. Note that if there is an open niche in the gut ecosystem, the escape-variant can simply replace the parental strain with no noticeable effect on total population size. Unless bacterial evolution is specifically examined, this can lead to the conclusion that SIgA has had no negative effect on the targeted pathogen.

### Escaping canonical SIgA

**Fig. 3:**
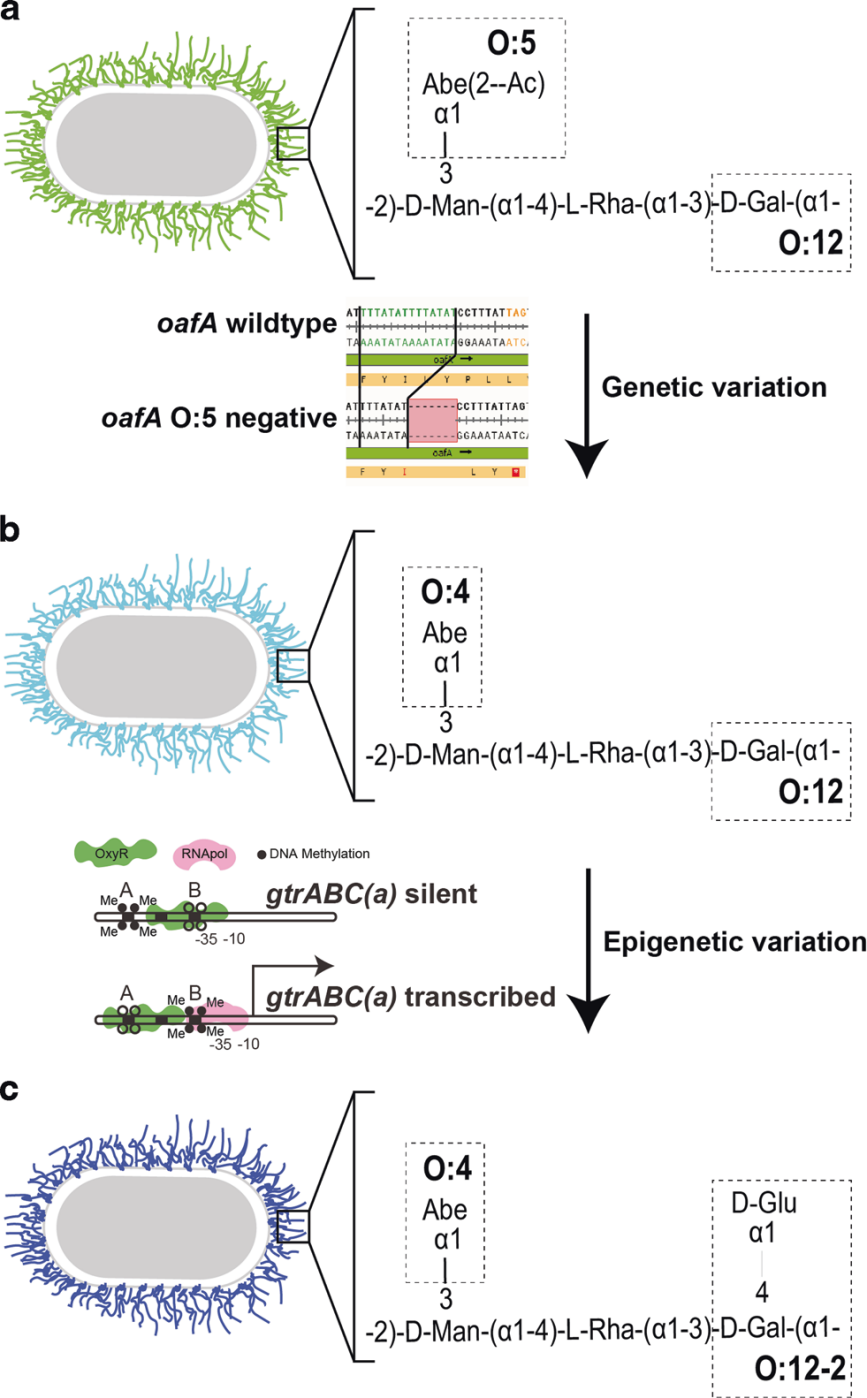
Observed *Salmonella* Typhimurium O-antigen variation under SIgA pressure. The wildtype *Salmonella* Typhimurium O-antigen is designated as O:4[5],12-0. Production of the O:5 epitope is abolished by contraction of a 7 bp microsattelite repeat in the *oafA* gene (**a**). Additionally the O:12-0 epitope (the unmodified backbone galactose) can be modified to O:12-2 (α-1-4-linked glucose added to the galactose residue) by upregulating expression of a *gtrABC* operon, controlled epigenetically (**b**) to produce the O:4,12-2 serovar (**c**)^26^.

The most obvious way to escape SIgA-mediated aggregation is to acquire mutations that alter or remove the targeted surface epitope(s). In murine non-Typhoidal *Salmonellosis*, protective SIgA targets the *Salmonella* O-antigen, whose structure is shown in Fig. 3a. We can directly observe overgrowth of IgA-escape *Salmonella* variants within 48 h of infection in a vaccinated mouse^26^, and equivalent mutations are frequently recovered from human and animal infections^102^. Analysis of the precise genetic/biochemical changes allowing *S*. Typhimurium to escape repeatedly identified two modifications:

1. Contraction of a 7 bp tandem repeat in the O-antigen abequose acetyltransferase (*oafA)*, leading to incorporation of non-acetylated abequose into the glycan repeat units (Fig. 3b).
2. Epigenetic regulation of a *gtrABC* operon encoding for a glucosyl transferase to turn on O-antigen glucosylation (Fig. 3c)^26^.

Each of these changes were sufficient to decrease the affinity of vaccine-induced SIgA for the *Salmonella* surface^26^. Therefore, selection for O-antigen modification by SIgA is strong and can occur via reproducible trajectories.

Strikingly, both *oafA* and *gtrABC* lie outside of the main O-antigen synthesis operon. On closer inspection, this makes some sense—most enzymes in the main O-antigen synthesis pathway are highly substrate-specific: change one, and you need corresponding mutations up and downstream in the pathway^103,104^. On the fitness landscape, that means that obtaining improved fitness via this route requires crossing a large number of deep valleys and is statistically unlikely (an exception being horizontal acquisition of a completely new O-antigen biosynthesis cluster, which seems to have occurred in rare cases^105^). Also striking is that both of these enzymes are in fact associated with degraded integrated phage genomes in the chromosome^83,106^. Temperate bacteriophages often carry such extra accessory genes (morons) that modify the primary receptors of the phage (often O-antigens or teichoic acids) and therefore make their hosts resistant to subsequent attack by related phages^107^. Intriguingly, these mechanisms seem to have undergone “exaptation” (i.e., repurposing) to provide resistance to SIgA-mediated clearance. Potentially this is much less surprising than it first appears. Both phage and SIgA need to bind to the bacterial surface to infect or aggregate bacteria, respectively. Therefore, mechanisms that help prevent phage binding are likely to also resist SIgA. As all bacterial species living in open systems are under continuous selection from bacteriophages, it follows that most will have at least a few co-optable traits to evade SIgA.

### Escaping enchained growth via alternative mechanisms

There are two alternative ways to escape SIgA enchained growth. The first is to produce abundant surface antigen that is only weakly bound to the bacterial surface. This fails to crosslink even with high-affinity antibodies as antibody will simply pull antigen off the surface^93,101^. While Lipopolysaccharide O-antigens are strongly anchored in the outer-membrane via the hexa-acylated lipidA moiety, most polysaccharide capsules are linked by only a single acyl chain. Extracellular polysaccharides are not linked at all.

A second, more surprising, option is to grow slowly. Enchained growth generates aggregates because two cells become cross-linked as they divide, and this mode of aggregation dominates as long as the total population density is low^84^. Each of those cross-linked daughter cells is also cross-linked as it divides, generating a chain of 4, and then 8 and then 16 cells and so on. However, this process is never perfect. Crosslinks tend to fall apart with a given half-life. If two daughter cells are cross-linked during division, but the crosslink falls apart before the next cell division, then we will never achieve a chain-length greater than two. This becomes particularly relevant when considering cross species reactive or polyreactive SIgA coating parts of the commensal microbiota^108^. Even if a pathogen-induced SIgA cross-reacts with a beneficial gut microbe, SIgA-driven enchained growth will have minimal to no impact on the beneficial microbe as long as the IgA-microbe interaction includes weak links (either the antibody is low-affinity or the antigen is sparse and/or weakly cell surface-linked), and/or the growth rate of the microbe remains slow. Rapid growth is associated with dysbiosis in the gut, leading to one strain dominating the community, and it is this “pathogen-associated behavior” that particularly strongly drives the negative selective effects of SIgA-mediated enchained growth^93,101^.

### Enchained growth can suppress the rate of adaption

In contrast to inflammation, which tends to increase the supply of mutations and the rate of horizontal gene transfer, vaccine-induced SIgA suppresses this. Oral-vaccine-induced SIgA prevents pathogen-induced inflammatory responses that would otherwise promote latent bacteriophage mobilization^79^. Moreover, enchained growth generates bacterial aggregates containing a very limited number of clones, physically inhibiting contact-mediated plasmid transfer, and ensuring that whole clonal lineages are eliminated en bloc^84^. This population structure biases the unit of selection to whole enchained clonal lineages, rather than individual bacteria. The corresponding major reduction in the effective population size increases the strength of genetic drift^20,84,93^. Therefore, while the selective pressure exerted by high-affinity specific SIgA can be very strong, the chance to randomly produce mutants with higher fitness is actually suppressed by SIgA^93^.

### Positive selection by SIgA?

Confusingly, there is also good evidence of positive fitness effects of canonical SIgA binding on bacterial species in the gut^109,110^. Donaldson et al. showed that capsular polysaccharide antigen-specific SIgA is necessary to generate *Bacteroides fragilis* aggregates in the colonic mucus^109^. In the absence of SIgA-bacterial-capsule interactions, this niche remained unstable and could be invaded by novel strains. Plausibly, this could be due to enchained growth and/or mucus cross-linking allowing microcolonies to spread ever deeper into the mucus and thus oppose the outward movement and sloughing of the mucus layer. It should be noted that this is a relatively small effect compared to *Salmonella* luminal effects described above, involving the stability of around 0.01% of the total luminal *B.fragilis* population (10^6^ members of a 10^10^ bacteria total population)^109^. Nevertheless, this suggests that SIgA may actually be beneficial to mucus-resident pathogens. Studies in IgA-deficient mice suggested that IgA rather promotes *Helicobacter* colonization of the stomach mucosa^111^. *Helicobacter hepaticus* also seems to drive colitis in IL-10-deficient mice despite high specific SIgA production^112^. However, studies with oral vaccination report some protection against *Helicobacter* which is at least partly attributable to SIgA^113–116^. This could be consistent with high-affinity SIgA blocking the initial phases of mucus colonization by *Helicobacter* if colonization occurs after vaccination, but promoting mucus colonization when SIgA is only induced post-colonization. Further work is needed to fully understand these phenomena.

To summarize, SIgA has a subtler and more precise effect on the fitness landscapes of the intestine than inflammatory responses (Fig. 1), based on its ability to exert very specific selective pressures and to suppress evolvability. This perhaps explains why non-inflammatory secretory antibodies seem to have evolved twice independently during vertebrate evolution^117^: They are safe and precise tools for controlling the abundance of unwanted organisms, and perhaps promoting the abundance of required organisms, without major perturbations to the healthy surrounding microbiota.

## Mapping evolutionary trajectories for mucosal vaccine design

As discussed above, “neutralization” of a bacterial pathogen, i.e., reducing its niche to nothing, is rarely possible. Where it is, these epitopes often show high levels of strain-level diversity, making them challenging to universally target with vaccination^118^. But if general surface-targeting antibodies rapidly select for immune escape, then surely this approach is also doomed? Here there is hope based on the concept of “Evolutionary Traps”^26^. Specific SIgA generates a defined shift in fitness maxima, forcing the targeted bacterium to evolve towards a new equilibrium. With current analytic capabilities, we have the possibility to play cat-and-mouse with the vaccine and bacterial evolution. In order to identify the most likely evolutionary trajectories, we can start with a whole-cell inactivated oral vaccine constructed from the wild-type bacterial strain. We then challenge the vaccinated animal, and via analysis of the emerging bacteria can identify strains with increased fitness in vaccinated hosts. Via biochemical and genetic techniques, we can identify surface epitope changes responsible for the increased fitness, i.e., changes allowing the bacterium to attain a new maximum fitness in vaccinated animals^26^.

This information can then be used to design the second generation of vaccines, which should cut off the evolutionary trajectory both to the original fitness maximum, and to this new fitness maximum. While there may be several possible ways for a bacterium to modify its surface without losing overall fitness, the evolutionarily feasible possibilities are not infinite. At some point we expect to steer the evolutionary trajectory of our pathogen towards fitness maxima that involve an evolutionary trade-off. In the case of *S*. Typhimurium SL1344, this is possible with a tetravalent oral vaccine, which forces the positive selection of clones carrying spontaneous deletion of the *wzyb/rfc* gene. These bacteria cannot polymerize their O-antigen and therefore present with a semi-rough phenotype, associated with increased susceptibility to Complement, bile acids, common environmental phages and detergents^26^. They are therefore poor survivors in tissues and poor in transmission to new hosts. An interesting feature of these observations is the high level of reproducibility of the observations: why does *S*. Typhimurium SL1344 not have more potential escape options? The kinetics of serovar replacement in well-defined populations such as USA cattle ranches^119^ suggests that this limited escape potential is a detriment for the individual serovars. One hypothesis is the existence of a trade-off between the number of potentially costly IgA-escape mechanisms integrated into the genome, which are often phage-genome associated^120^, and the pressure to escape immunity.

The “Evolutionary Trap” concept can be taken one step further. Effectively, we can understand SIgA as a tool to manipulate niche competition in the gut lumen. It logically follows that we can design “probiotic” niche-competitors, which when combined with oral vaccination can generate complete exclusion of the targeted pathogen/pathobiont. As SIgA-binding generates a fitness disadvantage for the invading pathogen, then a niche-competitor strain that is not IgA-bound will have a higher probability to outcompete^121^. Using a modified *S*. Typhimurium as a benign niche competitor, combined with a pathogen-specific oral vaccine, we can generate sterilizing immunity against pathogenic *S*. Typhimurium in the gut lumen of mice^121^.

A question then arises of whether this mechanism explains why live-attenuated vaccines generate better protection than inactivated oral vaccines. A live vaccine which colonizes for several weeks has the potential to go through cycles of antibody induction and selection, tracking the expected evolution of the pathogen. Moreover, persistent vaccine strains can clearly behave as a niche competitor to the pathogen^122^. However, we suggest that this is not the whole story. Live *Salmonella* vaccines induce both trained immunity and effector T cell responses^123,124^ and fully non-replicative live *Salmonella* vaccine also provided superior protection to killed oral vaccines in a murine model^125^. On the other side, for human translation a major requirement of live vaccines is that they do not chronically colonize the host^125,126^. Vaccine reversion to virulence and disease in immunocompromised hosts remains a major concern, most prominently demonstrated by the live-attenuated oral Polio vaccine^127,128^. Correspondingly, new live-attenuated vaccines are specifically being developed to minimize the duration of colonization, and these will be associated with a lower probability to spontaneously generate an SIgA “evolutionary trap” response. It would therefore be beneficial to build “Evolutionary Trap” oligoclonal versions of these next-generation live vaccines.

## Conclusions and outlook

Here we have examined two situations: (1) in which acute inflammation drives global shifts in the intestinal environment, typically benefiting facultative anaerobes and increasing the rate of evolution; and (2) considering the fitness consequences of secretory IgA. The mechanisms involved are divergent and can operate simultaneously. They reveal a close intertwining of the evolution of bacteria, bacteriophages and the mucosal immune system. But what is central here, is that understanding these mechanisms potentially allows us to work *with* rather than against the evolution of intestinal microbes^26^.

It has many times been noted that had Darwin been a physicist, we would call it “the law of evolution”. Evolution is a ubiquitous force that underlies or affects every observation we make in biology. In the gut, where bacterial (and bacteriophage) loads are incredibly high^129^, and all those cells (and viruses) are actively replicating, then we are facing the evolutionary equivalent of a juggernaut with the gas pedal flat down. Microbial evolution gets a bad rap, driving antimicrobial resistance, immune escape and generating zoonoses that cross species barriers. But it is high time we learned to harness this force for good. “Evolutionary trap” approaches should be robust to resistance selection because the evolutionary trade-off is a local fitness maximum. This has the potential to generate herd-immunity for intestinal pathogens, either by decreasing the abundance of fully virulent pathogens being shed to the environment, or by reducing the size of infectious reservoirs per se. As with our current childhood vaccination schedule, this would provide protection not only to the effectively vaccinated, but also to immunocompromised individuals who are particularly susceptible to infection. Proof-of-concept for “evolutionary trap” vaccines exists for non-Typhoidal *Salmonellosis* in mice, and already the concept has been co-opted to work for viruses^130^.

We still often focus on bacterial population sizes (16S amplicon sequencing, bacterial plating) to analyze the effects of immunity in the gut, which often overlooks bacterial evolution. If we really want to steer bacterial evolution to our benefit, we need a robust understanding of the selective pressures at play across multiple interventions and systems. Immunity is only one of many influences on the gut fitness landscape, which include diet, pharmaceuticals, gut physiology (and circadian rhythm) and evolution of (and immigration into) the gut microbiota itself. The potential effect of a mucosal immune intervention will depend on its relative strength compared to all other determinants. Detailed further research, both in reductionist systems and in clinical settings is urgently needed to address this. For example, we need to understand how conserved and reproducible the shifts in metabolic niche are during different perturbations and with differing microbiota compositions. We also need better tools for induction of intestinal SIgA against diverse and defined bacterial surface structures, as well as a better understanding of how the mucosal immune system works in humans and livestock. For example, human IgA deficiency is relatively common, but compensated by secretory IgM, a feature which is not replicated in the gut of IgA-deficient mice^84^. It remains unclear if human SIgM can also generate Evolutionary Traps in the gut, but this appears theoretically possible.

Overall, compared to the hunt for new antibiotics, the hunt for oral vaccines that generate immunity-driven Evolutionary traps has a huge advantage: Resistance *is* futile.
